# Synthesis, Spectral and Antibacterial Studies of Copper(II) Tetraaza Macrocyclic Complexes

**DOI:** 10.3390/ijms13044982

**Published:** 2012-04-19

**Authors:** Puchakayala Muralidhar Reddy, Rondla Rohini, Edulla Ravi Krishna, Anren Hu, Vadde Ravinder

**Affiliations:** 1 Department of Chemistry, Kakatiya University, Warangal 506 009, A.P, India; E-Mails: pmdreddy@gmail.com (P.M.R.); prmnreddy@gmail.com (R.R.); edulla2008@gmail.com (E.R.K.); 2 Department of Chemistry, National Dong Hwa University, Hualien 974, Taiwan; 3 Department of Laboratory Medicine and Biotechnology, Tzu Chi University, Hualien 97004, Taiwan

**Keywords:** synthesis, antibacterial studies, tetraaza macrocycles, copper(II) complexes

## Abstract

A novel family of tetraaza macrocyclic Cu(II) complexes [CuLX_2_] (where L = N_4_ donor macrocyclic ligands) and (X = Cl^−^, NO_3_^−^) have been synthesized and characterized by elemental analysis, magnetic moments, IR, EPR, mass, electronic spectra and thermal studies. The magnetic moments and electronic spectral studies suggest square planar geometry for [Cu(DBACDT)]Cl_2_ and [Cu(DBACDT)](NO_3_)_2_ complexes and distorted octahedral geometry to the rest of the ten complexes. The biological activity of all these complexes against gram-positive and gram-negative bacteria was compared with the activity of existing commercial antibacterial compounds like Linezolid and Cefaclor. Six complexes out of twelve were found to be most potent against both gram-positive as well as gram-negative bacteria due to the presence of thio group in the coordinated ligands.

## 1. Introduction

Macrocyclic ligands are considerably attractive in the quest for new chemistry, because they offer a wide variety of donor atoms, ionic charges, coordination numbers and geometry of the resultant complexes [1–5]. The understanding of the metal ion chemistry of macrocyclic ligands has important implications for a range of chemical and biochemical applications [6,7]. Many metal complexes of naturally occurring porphyrins, corrins and phthalocyanines have been investigated because of their potential as dyestuffs or pigments [8–11]. Macrocyclic ligand complexes are of great importance in enhancing various industrial applications and in a number of biological processes such as photosynthesis and dioxygen transport [12] catalytic properties, potential applications as metal extractants, radiotherapeutic and medical imaging agents and to potency towards DNA binders [13] with a high potential in anti-tumor therapy, has provided a motivation for investigation of the metal ion chemistry of these systems, as well as of cyclic ligand systems [8,9,14,15]. Cu(II) is the most studied metal ion among all the transition metal ions [16,17]. Cu(II) complexes are known to play a significant role either in naturally occurring biological systems or as pharmacological agents [18,19]. In connection with previous investigations [1,2,20], on the coordinating properties of tetraaza macrocycles, and in order to isolate new transition metal complexes with potential antimicrobial properties, we have studied the synthesis, spectroscopic and biochemical aspects of tetraaza macrocyclic complexes of copper(II) derived from *o*-phthalaldehyde with various diamines.

## 2. Results and Discussion

A series of twelve tetraaza macrocyclic Cu(II) complexes were synthesized by treating CuCl_2_·2H_2_O and Cu(NO_3_)_2_·3H_2_O with the six macrocyclic Schiff base ligands. All the complexes are stable to the atmosphere. The complexes are soluble in chloroform, DMSO, DMF and aqueous methanol. The elemental analyses (Table 1) are consistent with the proposed structure of the complexes. The molar conductance values of the complexes **1.5** and **2.5** in DMF at room temperature (65.0 and 68.3 ohm^−1^·cm^2^·mol^−1^) confirm their 1:2 electrolytic natures and the low molar conductance values of other complexes indicate non-electrolytic nature.

### 2.1. Infrared Spectral Data

The main bands and their assignments are listed in Table 2. In the IR spectra of macrocyclic Cu(II) complexes, a medium intensity band due to υ_(C=N)_ was shifted towards lower side about 20–33 cm^−1^ compared to the ligand spectra and was appeared in the range of 1598–1573 cm^−1^ [21]. The appearance of a lower intensity band in the region of 524–505 cm^−1^ corresponds to the υ_(M–N)_ vibration supports the fact that the ligands coordinate to the metal ions through the nitrogen of C=N group in all the complexes [22–24]. A band present in the range of 318–302 cm^−1^ in the spectra of **1.1**, **1.2**, **1.3**, **1.4** and **1.6** complexes indicating the presence of two chlorides in trans position around copper center [17,25]. The presence of chloride ions in **1.1**, **1.2**, **1.3**, **1.4** and **1.6** complexes are detected by the addition of silver nitrate reagent leading to the formation of white precipitate. However, in the case of complex **1.5,** this band is not observed indicating chloride ions are not in the coordination sphere. The coordination of the nitrate groups have been confirmed in the complexes **2.1**, **2.2**, **2.3**, **2.4** and **2.6** by the band in the region of 230–240 cm^−1^ may be assigned [9] to υ_(M–O)_ of the ONO_2_ group [26]. The additional bands observed around ~1410, 1300 and 1020 cm^−1^ [25] were obtained in the spectra of the nitrato complexes, which consistent [26] with the monodentate nature of the nitrato group [25,27,28]. The absence of these bands was observed in case of complex **2.5** indicating nitrate groups are not in the coordination sphere. The macrocyclic Cu(II) compounds (**1.2** and **1.6**) contain a broad band in the region 3505– 3374 cm^−1^ due to the presence of lattice water molecules[18,22].

### 2.2. EPR Spectral Data

The EPR spectra of the Cu(II) complexes have been recorded at room temperature as well as at liquid nitrogen temperature and their g_ll_ and g_⊥_ values have been calculated. The observed data show that g_ll_ = 2.085–2.219 and g_⊥_ = 2.018–2.046 (Table 3). The values of g_ll_ and g_⊥_ are closer to 2 and g_ll_ > g_⊥_. This suggests that the unpaired electron is in the d_x2-y2_ orbital and hence, ^2^B_1_ is the ground state. It should be noted that for an ionic environment g_ll_ > 2.3, while for a covalent environment g_ll_ < 2.3. The copper complexes show g_ll_ < 2.3 indicating their considerable covalent character [9,29]. The g values are related by the expression G = (g_ll_ − 2)/(g_⊥_ − 2) which suggests an exchange interaction between copper centers in the polycrystalline solid. According to Hathway [30] if G > 4, the exchange interaction is negligible. In the present case, the axial symmetry parameter, G, lies in the range 4.025–5.896, which indicates no considerable exchange interaction in these solid complexes.

### 2.3. Electronic Spectra and Magnetic Data

The magnetic moment of all the Cu(II) complexes at room temperature lie in the range of 1.86–1.98 B.M. given in Table 1, corresponding to one unpaired electron. This indicates that these complexes are monomeric in nature and the absence of metal-metal interaction [17]. The electronic spectra of the complexes [Cu(L)X_2_](X = Cl^−^, NO_3_^−^) display two characteristic bands in the region 21226–22801 cm^−1^ and 16008–18348 cm^−1^ these may be assigned to ^2^B_1g_→^2^E_g_ and ^2^B_1g_→^2^A_1g_ transitions respectively. The third band assigned to ^2^B_1g_→^2^B_2g_ transition band is usually not observed as a separate band in the tetragonal field. Therefore, it may be concluded that all the [Cu(L)X_2_] complexes show distorted octahedral structure (Scheme) [31]. The complexes [Cu(L)]X_2_ display broad band in the region 18181–18457 cm^−1^ and 13065–13524 cm^−1^ corresponding to transitions ^2^B_1g_→^2^E_g_ and ^2^B_1g_→^2^A_1g_ which suggests the square planar geometry [19].

### 2.4. Thermal Analysis

Thermograms of TGA and DTA of macrocyclic Cu(II) compounds were recorded in nitrogen atmosphere at a heating rate of 10 °C/min. In the thermogram of complexes [Cu(TBACD)Cl_2_]·2H_2_O (**1.2**) and [Cu(TBAHD)Cl_2_]·2H_2_O (**1.5**), the initial weight loss of 15.36% (Calcd 15.28%) and 14.45% (Calcd 14.37%) at 70–110 °C corresponding to loss of two lattice held water molecules [18,22]. This fact was further supported by their DTA curves, which contain endothermic peaks in the temperature range 90–125 °C. The TGA decomposition curve, a peak corresponding to the loss of organic moiety in the temperature range of 238–252 °C was observed. On the other hand, the thermograms of other macrocyclic Cu(II) compounds showed only a single decomposition curve in the region 220–260 °C corresponding to the loss of organic moiety. Above 455 °C, organic moieties in macrocyclic Cu(II) compounds were decomposed leading to the formation of copper oxide.

### 2.5. Mass Spectral Analysis

In the mass spectra of respective macrocyclic Cu(II) complexes, molecular ion peaks, were observed at different *m*/*z* (M^+^/[M + Na]^+^) values. The molecular ion peaks and isotopic pattern of Cu(II) complexes shows different *m*/*z* values with different intensities. The mass spectra contain molecular ion peaks at *m*/*z* 511 (M^+^, complex **1.1**), 517 (M^+^, complex **1.2**), 507 (M^+^, complex **1.3**), 543 (M^+^, complex **1.4**), 483 (M^+^, complex **1.5**), 517 (M^+^, complex **1.6**), 563 (M^+^, complex **2.1**), 586 ([M + Na]^+^, complex **2.1**), 533 (M^+^, complex **2.2**), 560 (M^+^, complex **2.3**), 583 ([M + Na]^+^, complex **2.3**), 595 (M^+^, complex **2.4**), 535 (M^+^, complex **2.5**), and 565 (M^+^, complex **2.6**). This data is in good agreement with the respective molecular formulae.

**Scheme 1:**
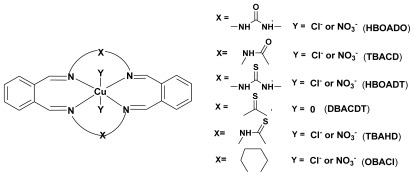
Representative structures of copper complex.

Based on the physicochemical and the spectral studies the tentative structures proposed for the complexes are shown in Scheme 1.

### 2.6. Biological Results and Discussion

**Figure 1:**
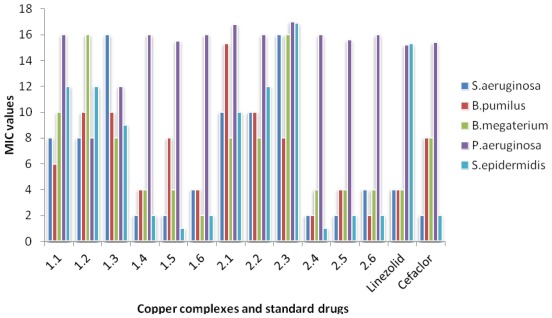
Comparison of MIC values (in μg/mL) of Cu(II) macrocyclic complexes and standard drugs against different bacteria.

Twelve chemically synthesized Cu(II) macrocyclic complexes were tested *in vitro* for their antibacterial activity against five test bacteria namely *S. aeruginosa*, *S. epidermidis*, *B. pumilus*, *B. megaterium* and *P. aeruginosa.* The minimum inhibitory concentrations of complexes were determined by liquid dilution method. The minimum inhibitory concentration at which no growth was observed was taken as the MIC values. All the complexes of the tested series possessed good antibacterial activity against both gram-positive bacteria and gram-negative bacteria. The higher antibacterial activity of the copper complexes is may be due to the change in structure due to coordination and chelating tends to make metal complexes act as more powerful and potent bactereostatic agents, thus inhibiting the growth of the bacteria. Six complexes out of twelve (**1.4**, **1.5**, **1.6**, **2.4**, **2.5** & **2.6**) were most potent against both gram-positive as well as gram-negative bacteria. However, complexes **1.1**, **1.2**, **1.3**, **2.1**, **2.2** & **2.3** were also showed noticeable MIC ranging from 1–16 μg/mL against *S. aeruginosa*, *B. pumilus* and *B. megaterium* (Table 5). The gram positive were much more susceptible to the series as compared to gram-negative bacteria. The twelve Cu (II) complexes were also compared with two commercial antibiotics namely Linezoid and Cefaclor. The six complexes **1.4**, **1.5**, **1.6**, **2.4**, **2.5** & **2.6** registered better antibacterial in comparison with commercial antibiotics. The activity of these complexes is due to the presence of thio group in the coordinated ligands. Comparison of MIC values (in μg/mL) of Cu(II) macrocyclic complexes and standard drugs against different bacteria are given in Figure 1.

## 3. Experimental Section

### 3.1. Materials and Methods

The metal salts CuCl_2_·2H_2_O and Cu(NO_3_)_2_·3H_2_O were purchased from E. Merck. All the chemicals used were of AR grade and were procured from Aldrich. All solvents used were of AR grade. Six macrocyclic ligands viz. 7,8,9,18,19,20-hexahydrodibenzo[*g*,*p*] [1,2,4,5,10,11,13,14]- octaazacyclooctadecine-8,19-dione [HBOADO], 7,8,17,18-tetrahydrodibenzo[*f*,*n*] [1,2,4,9,11,12]- hexaazacyclohexadecine-8,17-dione [TBACD], 7,8,9,10,19,20,21,22-octahydrodibenzo[*c*,*m*] [1,6,11,16]-tetraazacycloicosine [OBACI], 7,8,9,18,19,20-hexahydrodibenzo[*g*,*p*] [1,2,4,5,10,11,13,14]-octaazacyclooctadecine-8,19-dithione [HBOADT], 7,16-dihydrodibenzo[*e*,*l*] [1,3,8,10]-tetraazacyclo tetradecine-7,16-dithione [DBACDT] and 7,8,17,18-tetrahydrodibenzo[*f*,*n*] [1,2,4,9,11,12]-hexaazacyclohexadecine-8,17-dithione [TBAHD] were synthesized according to the procedure reported by shanker *et al.* [32]. Gram positive microorganisms like *Staphylococcus aeruginosa* (ATCC-29213), *Staphylococcus epidermidis* (MTCC-2639), *Bacillus pumilus* (MTCC-1456), *Bacillus megaterium* (MTCC-428) and gram negative microorganism like *Pseudomonas aeruginosa* (ATCC-27853) from IMTECH, Chandigarh were used for antibacterial studies.

### 3.2. Measurements

The elemental analyses were obtained by using a Perkin-Elmer-2400 CHN elemental analyzer. The complexes were analyzed for Cu(II) content gravimetrically by literature procedures (Vogel AI (1989) Vogel’s text book of quantitative chemical analysis, 5th edition Longman, Amsterdam) after decomposing the organic matter with a mixture of HNO_3_ and HCl and evaporating the residue to dryness with concentrated H_2_SO_4_. UV-Vis spectra were recorded on Shimadzu UV-160A spectrophotometer, EPR spectra were recorded on Varian E-112X-Q band EPR Spectrometer. IR spectra in KBr pellets on Perkin-Elmer-283 spectrophotometer. LC-ESI MS was used to obtain mass spectra. Gouy balance calibrated with Hg[Co(NCS)_4_] was used for the determination of magnetic susceptibilities of complexes in solid state at room temperature. Thermograms were recorded on mettler-TA-2000C model. TG-DSC equilibrated at 25 ± 0.05 °C. The electrical conductivities of 10^−3^ M solutions in dichloromethane were performed using Digisun Digital conductivity meter model DL-909.

### 3.3. Synthesis of Dichloro/Nitrato Cu(II) Complexes [CuLX_2_]

To a stirred solution of the respective copper salts (0.519 g, 0.003 mol) dissolved in methanol (~20 mL) in a round-bottomed flask, methanolic solution (~25 mL) of ligand (0.003 mol., viz. 1.17 g of HBOADO, 1.038 g of TBACD, 1.116 g of OBACI, 1.26 g of HBOADT, 1.044 g of DBACDT, 1.134 g of TBAHD respectively) were added with the help of a dropping funnel. The reaction mixture was stirred magnetically. The resulting solution was concentrated to 5 mL under reduced pressure and a few mL of diethyl ether was added to initiate the crystallization. The resulting precipitate was separated by suction filtration, washed several times with diethyl ether, dried *in vacuo* and was recrystallized using dichloromethane and diethyl ether solvent mixture.

## 4. Conclusions

In the present communication, we have synthesized and characterized twelve copper (II) complexes containing tetraaza macrocyclic ligands. Based on the analytical and spectral data, we assume that the complexes (**1.5** & **2.5**) are square planar and the rest of the complexes show distorted octahedral geometry. All the complexes of the tested series were found to have good antibacterial activity. Complexes **1.4**, **1.5**, **1.6**, **2.4**, **2.5** & **2.6** were most potent against both gram-positive as well as gram-negative bacteria due to the presence of thio group in the coordinated ligands.

## Figures and Tables

**Table 1:** Physical, analytical and electronic spectral data of macrocyclic Cu(II) complexes.

Comp. No.	Cu(II) Compound/Molecular Formula	Λ_M_	μeff (B.M.)	λ_max_ (cm^−1^)	Analyses (%) Found (Calculated)			

					C	H	N	Cu
1.1.	[Cu(HBOADO)Cl_2_] C_18_H_16_Cl_2_N_8_O_2_Cu	16.2	1.96	16008, 21226	42.38 (42.32)	3.18 (3.16)	22.04 (21.94)	12.36 (12.44)
1.2.	[Cu(TBACD)Cl_2_]·2H_2_O C_18_H_18_Cl_2_N_6_O_4_Cu	14.0	1.98	17348, 22801	41.33 (41.83)	3.52 (3.51)	16.23 (16.26)	12.25 (12.30)
1.3.	[Cu(OBACI)Cl_2_] C_24_H_28_Cl_2_N_4_Cu	13.5	1.96	17857, 21988	56.88 (56.86)	5.57 (5.57)	11.02 (11.05)	12.56 (12.53)
1.4.	[Cu(HBOADT)Cl_2_] C_18_H_16_Cl_2_N_8_S_2_Cu	12.4	1.94	17847, 21255	40.01 (39.82)	2.84 (2.97)	20.72 (20.64)	11.55 (11.70)
1.5.	[Cu(DBACDT)]Cl_2_ C_18_H_12_Cl_2_N_4_S_2_Cu	65.0	1.91	13065, 18181	44.42 (44.77)	2.68 (2.50)	11.72 (11.60)	13.07 (13.16)
1.6.	[Cu(TBAHD)Cl_2_]·2H_2_O C_18_H_18_Cl_2_N_6_S_2_Cu	16.5	1.92	17699, 22527	41.95 (41.82)	3.50 (3.51)	16.22 (16.26)	12.24 (12.29)
2.1.	[Cu(HBOADO)(NO_3_)_2_] C_18_H_16_N_10_O_8_Cu	15.3	1.93	16422, 21712	38.23 (38.34)	3.02 (2.86)	24.81 (24.84)	11.34 (11.27)
2.2.	[Cu(TBACD)(NO_3_)_2_] C_18_H_14_N_8_O_8_Cu	16.1	1.89	17758, 22954	40.42 (40.49)	2.84 (2.64)	21.10 (20.99)	11.87 (11.90)
2.3.	[Cu(OBACI)(NO_3_)_2_] C_24_H_28_N_6_O_6_Cu	14.8	1.94	18201, 22147	51.48 (51.47)	5.05 (5.04)	15.06 (15.01)	11.38 (11.35)
2.4.	[Cu(HBOADT)(NO_3_)_2_] C_18_H_16_N_10_S_2_O_6_Cu	12.9	1.86	18348, 21875	36.24 (36.27)	2.68 (2.71)	23.42 (23.50)	10.72 (10.66)
2.5.	[Cu(DBACDT)](NO_3_)_2_ C_18_H_12_N_6_S_2_O_6_Cu	68.3	1.89	13524, 18457	40.31 (40.34)	2.16 (2.26)	15.74 (15.68)	11.91 (11.86)
2.6.	[Cu(TBAHD)(NO_3_)_2_] C_18_H_14_N_8_S_2_O_6_Cu	15.4	1.93	17700, 22756	38.12 (38.20)	2.54 (2.49)	19.82 (19.80)	11.34 (11.23)

**Table 2:** Infrared spectral data of macrocyclic Cu(II) complexes.

Comp. No.	Cu(II) Compound	Selected IR Bands (cm^−1^)			

		υ_C=N_	υ_NH_	υ_Cu–N_	Anion Peaks
1.1.	[Cu(HBOADO)Cl_2_]	1578	3320	516	307
1.2.	[Cu(TBACD)Cl_2_]_2_·H_2_O	1575	3326	518	304
1.3.	[Cu(OBACI)Cl_2_]	1596	-	520	318
1.4.	[Cu(HBOADT)Cl_2_]	1590	3324	524	302
1.5.	[Cu(DBACDT)]Cl_2_	1598	-	506	-
1.6.	[Cu(TBAHD)Cl_2_]_2_·H_2_O	1585	3383	520	314
2.1.	[Cu(HBOADO)(NO_3_)_2_]	1580	3318	510	235
2.2.	[Cu(TBACD)(NO_3_)_2_]	1573	3325	515	230
2.3.	[Cu(OBACI)(NO_3_)_2_]	1595	-	518	240
2.4.	[Cu(HBOADT)(NO_3_)_2_]	1587	3326	520	230
2.5.	[Cu(DBACDT)](NO_3_)_2_	1595	-	505	-
2.6.	[Cu(TBAHD)(NO_3_)_2_]	1580	3379	518	235

**Table 3:** EPR spectral data of macrocyclic Cu(II) compounds.

Comp. No.	Cu(II) Compound	g_11_	g⊥	|g|_avg_	G
1.1.	[Cu(HBOADO)Cl_2_]	2.119	2.024	2.055	4.958
1.2.	[Cu(TBACD)Cl_2_]·2H_2_O	2.212	2.043	2.099	4.930
1.3.	[Cu(OBACI)Cl_2_]	2.118	2.021	2.053	5.619
1.4.	[Cu(HBOADT)Cl_2_]	2.219	2.039	2.099	5.615
1.5.	[Cu(DBACDT)]Cl_2_	2.220	2.046	2.104	4.782
1.6.	[Cu(TBAHD)Cl_2_]·2H_2_O	2.171	2.029	2.076	5.896
2.1.	[Cu(HBOADO)(NO_3_)_2_]	2.108	2.022	2.050	4.909
2.2.	[Cu(TBACD)(NO_3_)_2_]	2.177	2.040	2.085	4.425
2.3.	[Cu(OBACI)(NO_3_)_2_]	2.085	2.018	2.040	4.722
2.4.	[Cu(HBOADT)(NO_3_)_2_]	2.165	2.030	2.075	5.500
2.5.	[Cu(DBACDT)](NO_3_)_2_	2.161	2.040	2.080	4.025
2.6.	[Cu(TBAHD)(NO_3_)_2_]	2.110	2.019	2.049	5.789

**Table 5:** Minimum inhibitory concentration (MIC) of Cu(II) macrocyclic complexes against test bacteria.

Comp. No.	Macrocyclic Cu(II) Complexes	MIC (μg/mL)				

		Sa	Bp	Bm	Pa	Se
1.1.	[Cu(HBOADO)Cl_2_]	8	6	10	16	12
1.2.	[Cu(TBACD)Cl_2_]·2H_2_O	8	10	16	8	12
1.3.	[Cu(OBACI)Cl_2_]	16	10	8	12	9
1.4.	[Cu(HBOADT)Cl_2_]	2	4	4	16	2
1.5.	[Cu(DBACDT)]Cl_2_	2	8	4	<16	1
1.6.	[Cu(TBAHD)Cl_2_]·2H_2_O	4	4	2	16	2
2.1.	[Cu(HBOADO)(NO_3_)_2_]	10	<16	8	>16	10
2.2.	[Cu(TBACD)(NO_3_)_2_]	10	10	8	16	12
2.3.	[Cu(OBACI)(NO_3_)_2_]	16	8	16	>16	>16
2.4.	[Cu(HBOADT)(NO_3_)_2_]	2	2	4	16	1
2.5.	[Cu(DBACDT)](NO_3_)_2_	2	4	4	<16	2
2.6.	[Cu(TBAHD)(NO_3_)_2_]	4	2	4	16	2
	Linezolid	4	4	4	<16	<16
	Cefaclor	2	8	8	<16	2

Sa: *S. aeruginosa* (ATCC-29213); Bp: *B. pumilus* (MTCC-1456); Bm: *B. megaterium* (MTCC-428); Pa: *P. aeruginosa* (ATCC-27853); Se: *S. epidermidis* (MTCC-2639).
